# The Beneficial Effect of Anticoagulation in Aortic Bioprosthesis is Associated with its Size

**DOI:** 10.21470/1678-9741-2019-0144

**Published:** 2020

**Authors:** Victor Dayan, Paula Farachio, Maria Jose Arocena, Amparo Fernandez, Diego Perez, Gerardo Soca

**Affiliations:** 1 Instituto Nacional de Cirugía Cardíaca, Montevideo, Uruguay.

**Keywords:** Ischemic Attack, Transient, Bioprosthesis, Aortic Valve, Atrial Fibrilation, Stroke, Hemorrhage, Hospitalization

## Abstract

**Objective:**

To evaluate the clinical and echocardiographic outcomes in aortic valve replacement (AVR) patients with aortic bioprosthesis under oral anticoagulation (OA).

**Methods:**

Patients who underwent AVR with bioprosthesiswere prospectively enrolled. They were classified based on postoperative use of OA. Clinical and operative variables were collected. Echocardiographic and clinical follow-ups were performed two years after surgery. The primary outcome evaluated was change in transprosthetic gradient. Secondary outcomes analyzed were change in New York Heart Association (NYHA) class, major bleeding episodes, hospitalization, stroke, and transient ischemic attack.

**Results:**

We included 103 patients (61 without OA and 42 with OA). Clinical characteristics were similar among groups, except for younger age (76±6.3 vs. 72.4±8.1 years, P=0.016) and higher prevalence of atrial fibrillation (0% *vs*. 23.8%, *P*<0.001) in the OA group. Mean (21.4±10 mmHg *vs*. 16.8±7.7 mmHg, *P*=0.037) and maximum (33.4±13.7 mmHg *vs*. 28.4±10.2 mmHg, *P*=0.05) transprosthetic gradients were higher in patients without OA. Improvement in NYHA class was more frequent in patients with OA (73% *vs*. 45.3%, *P*=0.032). Major bleeding, stroke, and hospitalization were similar among groups. OA was the only independent predictor for improvement of NYHA class after multivariate logistic regression analysis (odds ratio [OR]: 5.9, 95% confidence interval [CI]: 1.2-29.4; *P*=0.028). Stratification by prosthesis size showed that patients with ≤ 21 mm prosthesis benefited from OA.

**Conclusion:**

Early anticoagulation after AVR with bioprosthesis was associated with significant decrease of transprosthesis gradient and improvement in NYHA class. These associations were seen mainly in patients with ≤ 21 mm prosthesis.

**Table:** 

Abbreviations, acronyms & symbols				
AF	= Atrial fibrillation		iEOA	= Indexed effective orifice area
AMI	= Acute myocardial infarction		LVEF	= Left ventricular ejection fraction
AVB	= Atrioventricular block		MVR	= Mitral valve replacement
AVR	= Aortic valve replacement		MVS	= Mechanical ventilatory support
AXC	= Aortic cross-clamp		NYHA	= New York Heart Association
BPVT	= Bioprosthetic valve thrombosis		OA	= Oral anticoagulation
BSA	= Body surface area		OR	= Odds ratio
CABG	= Coronary artery bypass grafting		PVD	= Peripheral vascular disease
CE	= Carpentier Edwards		RBC	= Red blood cells
CI	= Confidence interval		SD	= Standard deviation
COPD	= Chronic obstructive pulmonary disease		SVD	= Structural valve degeneration
CPB	= Cardiopulmonary bypass		TAVR	= Transcatheter aortic valve replacement
CT	= Computed tomography		TIA	= Transient ischemic attack
EOA	= Effective orifice area		TTEs	= Transthoracic echocardiograms
ICU	= Intensive care unit			

## INTRODUCTION

The use of bioprosthesis for aortic valve replacement (AVR) has increased over the last couple of years. This is mainly due to the improvement in bioprosthesis durability as well as the increased tendency to operate on elderly patients. The main advantage of bioprosthesis is the lack of long term oral anticoagulation (OA)^[1]^. Nonetheless, bioprosthetic valve thrombosis (BPVT) is increasingly being recognized as a potentially reversible cause for structural valve degeneration (SVD)^[1-3]^.

BPVT is present in 11% of bioprosthetic valves explanted because of prosthesis dysfunction^[1,2]^. This entity may be suspected in patients with increased mean aortic valve gradient and is confirmed with the use of different imaging modalities^[2,4,5]^. Data from several small case series as well as non-randomized observational studies suggest that warfarin may be beneficial as a first-line therapy for suspected BPVT^[1-5,6,7]^. Although OA has been shown to be useful in this setting, there is not data regarding its association with prosthesis size.

Based on the previous data, our hypothesis was that patients under OA after AVR would have lower gradients and therefore better functional New York Heart Association (NYHA) class than patients without OA and that this effect would be seen mainly in patients with smaller prosthesis. The aim of our study was to evaluate the clinical and echocardiographic outcomes of patients with aortic bioprosthesis with and without OA.

## METHODS

This is a prospective cohort-based study in which we included patients who underwent AVR and received a bioprosthesis from January 2013 until December 2016. Patients were divided according to the postoperative use of OA. Baseline and operative variables were extracted from the institution’s database.

The primary outcome was change in aortic gradient. Secondary outcomes analyzed were change in NYHA class, major bleeding episodes, hospitalization, stroke, and transient ischemic attack (TIA).

Operative mortality was defined as death within the first 30 days after surgery or during the index hospitalization.

Extended inotrope use was defined as inotrope use beyond 12 hours from surgery.

Clinical follow-up was performed at the time of echocardiographic evaluation and the following variables were recorded: NYHA class, previous bleeding, hospitalization, stroke, and TIA.

The institution’s review board approved the study and informed consent was given before the surgical procedure.

### Surgery

AVR was performed through a median sternotomy with cardiopulmonary bypass and aortic cross-clamp (crystalloid cardioplegia was used in every case). Aortic valve was removed, and the annulus was decalcified. Interrupted "U" polyester 2-0 sutures with pledgets were used to anchor the prosthesis.

OA was started 2-7 days after surgery. The indications for OA were previous atrial fibrillation (AF) (42.9%), postoperative AF (45.2%), mitral valve replacement (MVR) (7.1%), atrial thrombus (2.4%), and previous deep venous thrombosis (2.4%).

### Echocardiogram

Comprehensive transthoracic echocardiograms (TTEs) were performed in all patients before hospital discharge (baseline TTE) and at follow-up (follow-up TTE). Mean time of echocardiographic follow-up was similar between groups (2.05±1.02 and 2.02±1.00 years in non-OA and OA groups, respectively). All TTE examinations were conducted according to the American Society of Echocardiography guidelines^[8]^. The mean transprosthetic gradient was calculated by using the modified Bernoulli formula. The effective orifice area (EOA) of the prosthesis was calculated by using the continuity equation.

### Statistics

Continuous variables were expressed as mean±standard deviation. Categorical variables were expressed as absolute value (%). Comparison between groups was performed using *t*-test and chi-square test.

The predictive role of OA on the primary and secondary outcomes was evaluated using logistic regression. The following variables were independently tested and those with a *P*<0.2 were entered in the multivariate model (age, gender, hypertension, diabetes, smoking, previous AF, associated coronary artery bypass grafting, associated MVR, left ventricular ejection fraction, creatininemia, and OA).

## RESULTS

Clinical and echocardiographic prospective follow-ups were performed on 103 patients (61 without OA and 42 with OA) who agreed to participate in this study. Clinical characteristics were similar among groups, except for increased age (76±6.3 *vs.* 72.4±8.1 years, *P*=0.016) and increased incidence of AF (0% *vs*. 23.8%, *P*<0.001) in the OA group (Table 1).

**Table 1:** Patients' preoperative variables.

	No OA (N=61)	OA (N=42)	*P*-value
Age, years (SD)	72.4 (8.1)	76 (6.3)	0.016*
Smoking (%)	10 (16.4)	7 (16.7)	0.971
Hypertension (%)	47 (77)	35 (83.3)	0.437
Diabetes (%)	16 (26.2)	11 (26.2)	0.996
Stroke (%)	2 (3.3)	1 (2.4)	0.790
PVD (%)	1 (1.6)	0 (0)	0.404
COPD (%)	1 (1.6)	0 (0)	0.404
Endocarditis (%)	1 (1.6)	1 (2.4)	0.789
AMI (%)	3 (4.9)	1 (2.4)	0.513
AF (%)	0 (0)	10 (23.8)	<0.001*
NYHA III-IV (%)	15 (28.3)	15 (40.5)	0.226
BSA (m^2^)	1.9 (0.3)	1.8 (0.2)	0.08
Creatininemia (mg/dl) (SD)	0.90 (0.39)	1.12 (1.40)	0.323
LVEF (%)	58.1 (10.7)	56.7 (13.3)	0.935
Previous CABG (%)	4 (6.6)	0 (0)	0.091
Previous valve surgery (%)	2 (3.3)	2 (4.8)	0.702

AF=atrial fibrillation; AMI=acute myocardial infarction; BSA=body surface area; CABG=coronary artery bypass grafting; COPD=chronic obstructive pulmonary disease; LVEF=left ventricular ejection fraction; NYHA=New York Heart Association; OA=oral anticoagulation; PVD=peripheral vascular disease; SD=standard deviation.

* *P*<0.05.

Operative and postoperative outcomes did not differ among groups (Table 2), except for higher incidence of MVR in the OA group (11.9% *vs*. 0%, *P*=0.006). Type and size of bioprosthesis were similar between groups. No patient at either group suffered major bleeding episodes, hospitalization, stroke, or TIA.

**Table 2:** Operative and postoperative outcomes.

	No OA (N=61)	OA (N=42)	*P*-value
CPB time (min) (SD)	104 (45)	103 (34)	0.934
AXC time (min) (SD)	76 (32)	79 (31)	0.721
Prosthesis size (mm) (SD)	21.7 (1.8)	21.6 (1.9)	0.596
Prosthesis (%)			0.457
St Jude Epic	29 (47.5)	22 (52.4)	
Mosaic	9 (14.8)	4 (9.5)	
Mitroflow	2 (3.3)	0	
Hancock II	16 (26.2)	14 (33.3)	
Braile	3 (4.9)	0	
CE-Perimount	2 (3.3)	2 (4.8)	
MVR (%)	0	5 (11.9)	0.006*
Use of RBC (%)	4 (6.6)	6 (14.3)	0.193
Extended inotrope use (%)	38 (62.3)	24 (57.1)	0.6
Stroke (%)	3 (4.9)	1 (2.4)	0.513
TIA (%)	8 (13.1)	2 (4.8)	0.159
AVB (%)	6 (9.8)	3 (7.1)	0.634
Pacemaker (%)	2 (3.3)	0 (0)	0.236
ICU stay (days) (SD)	3.8 (4.2)	2.7 (2.9)	0.153
MVS (hours) (SD)	16.2 (20.4)	15.5 (17.9)	0.862
Bleeding (ml)(SD)	954 (816)	691 (577)	0.058

AVB=atrioventricular block; AXC=aortic cross-clamp; CE=Carpentier Edwards; CPB=cardiopulmonary bypass; ICU=intensive care unit; MVR=mitral valve replacement; MVS=mechanical ventilatory support; OA=oral anticoagulation; RBC=red blood cells; SD=standard deviation; TIA=transient ischemic attack.

* *P*<0.05.

Patients in the OA group received anticoagulation for a mean of 11.7±13.2 months. Warfarin was used in 25 patients (60%) and non-warfarin OA in 17 patients (40%).

Mean (21.4±10 mmHg *vs*. 16.8±7.7 mmHg, *P*=0.037) and maximum (33.4±13.7 mmHg *vs*. 28.4±10.2 mmHg, *P*=0.05) transprosthetic gradients were significantly higher in patients without OA. Indexed effective orifice area (iEOA) was similar among groups (0.79±0.77 cm^2^/m^2^ *vs*. 0.77±0.22 cm^2^/m^2^ in non-OA and OA groups, respectively; *P*=0.357). No other differences were found during echocardiographic evaluation (Table 3).

**Table 3:** Clinical and echocardiographic evolution.

	No OA (N=61)	OA (N=42)	*P*-value
Mean gradient (mmHg) (SD)	21.4 (10.0)	16.8 (7.7)	0.037*
Max. gradient (mmHg) (SD)	33.4 (13.7)	28.4 (10.2)	0.05*
iEOA (cm^2^) (SD)	0.79 (0.77)	0.77 (0.22)	0.357
Dimensionless index	0.43 (0.11)	0.42 (0.08)	0.774
LVEF (%)	59.6 (6.6)	57.6 (9.0)	0.186
Central leak (%)	5 (8.2)	1 (2.4)	0.216
NYHA (%)			0.032*
Increase	7 (13.5)	2 (5.4)	
No change	22 (41.5)	8 (21.6)	
Decrease	24 (45.3)	27 (73.0)	

iEOA=indexed effective orifice area; LVEF=left ventricular ejection fraction NYHA=New York Heart Association; OA=oral anticoagulation; SD=standard deviation.

* *P*<0.05.

Adequate evaluation of leaflet mobility was not possible since all echocardiograms were transthoracic.

**Fig. 1:**
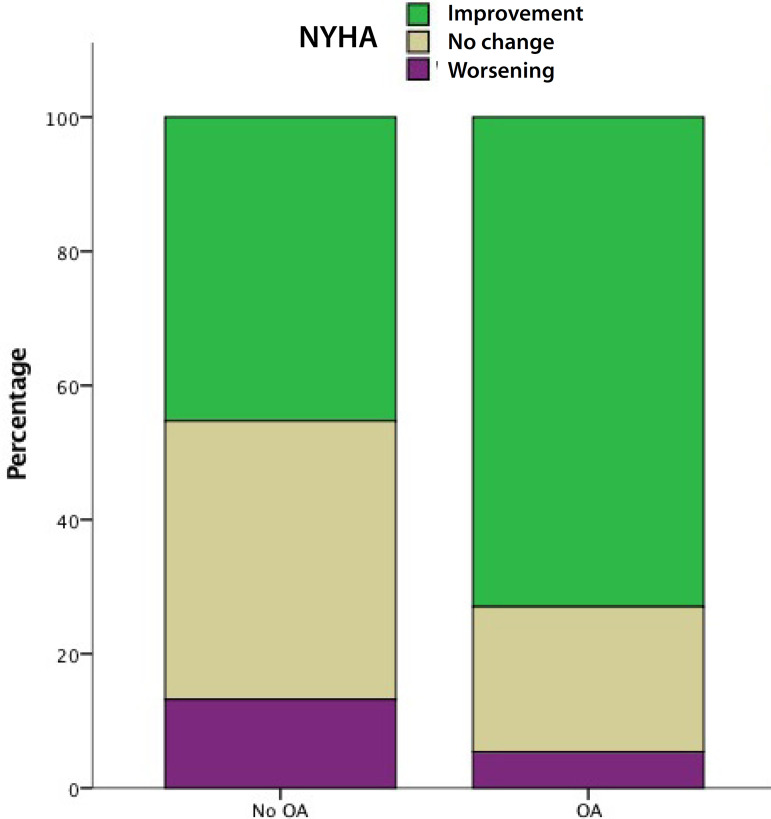
Change in New York Heart Association (NYHA) class at follow-up in patients with and without oral anticoagulation (OA). Green=patients who improved their NYHA; brown=patients who reported no change in their NYHA; purple=patients who reported worsening of their NYHA

Clinical evaluation revealed higher percentage in NYHA class improvement in patients with OA (73% *vs*. 45.3%, *P*=0.032) (Figure 1). Hemodynamic and clinical findings did not change after excluding patients who had concomitant MVR.

OA was the only independent predictor for NYHA class improvement after multivariate logistic regression analysis (odds ratio [OR]: 5.9, 95% confidence interval [CI]: 1.2-29.4; *P*=0.028).

**Fig. 2:**
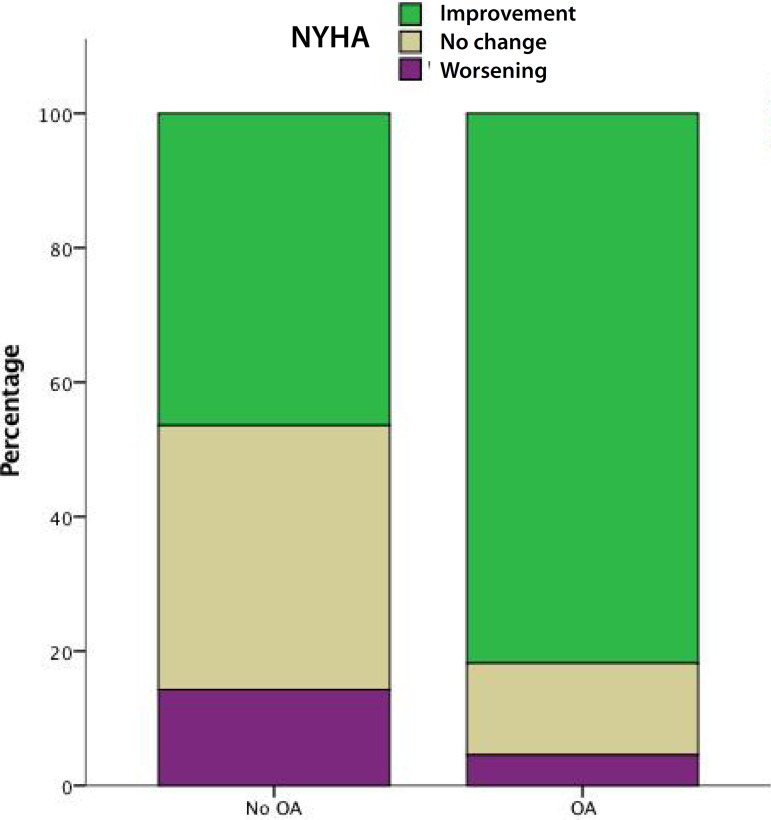
Change in New York Heart Association (NYHA) class at follow-up in patients with and without oral anticoagulation (OA) with ≤ 21 mm prosthesis. Green=patients who improved their NYHA; brown=patients who reported no change in their NYHA; purple=patients who reported worsening of their NYHA

**Fig. 3:**
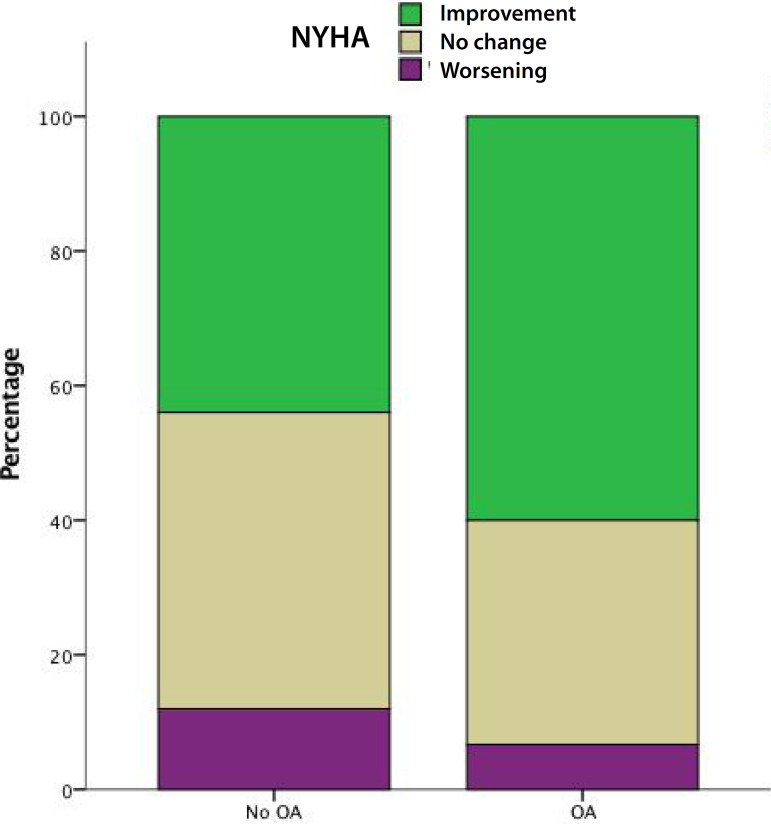
Change in New York Heart Association (NYHA) class at follow-up in patients with and without oral anticoagulation (OA) with > 21 mm prosthesis. Green=patients who improved their NYHA; brown=patients who reported no change in their NYHA; purple=patients who reported worsening of their NYHA

From the overall cohort, 48 patients (19 with OA and 29 without OA) received a ≥ 23 mm prosthesis and 55 patients (23 with OA and 32 without OA) received a ≤ 21 mm prosthesis. After stratifying patients according to bioprosthesis size, we found out that mean gradient (17.2±7.6 mmHg *vs*. 23.9±12.2 mmHg; *P*=0.05) and NYHA functional class (81.8% *vs*. 46.4%; *P*=0.01) improvement with OA occurred only in patients who received ≤ 21 mm bioprosthesis (Figures 2 and 3).

## DISCUSSION

OA in patients who received aortic bioprosthesis was associated with lower aortic gradient and was found to be the only independent predictor for improvement in NYHA class after AVR. Although evaluation of leaflet mobility was not possible, we were able to find an association between OA and clinical improvement, probably due to the lower aortic gradient. Similar iEOA between both groups of patients suggests that the higher gradient may be due to alterations in leaflet mobility, which have been described in patients with subclinical bioprosthetic thrombosis^[2,6]^. Our preliminary data suggest that the benefit of OA after AVR may be restricted mainly to patients with ≤ 21 mm biosprosthesis. The latter has not been previously reported.

Although symptomatic thrombosis represents the extreme end of the spectrum of BPVT and is probably underreported (prevalence of 1-2%), subclinical leaflet thrombosis with no associated symptoms is more frequent^[2,5,6]^. Reduced leaflet motion detected with high-resolution computed tomography (CT) in bioprosthetic aortic valves has been attributed to subclinical leaflet thrombosis^[2,9,10]^, which is associated with higher gradients, irrespective of iEOA.

Chakravarty et al.^[2]^ showed that in among the 55% of patients with reduced leaflet motion who had follow-up imaging, anticoagulation for three months was associated with restoration of normal leaflet motion in 36 (100%) of 36 patients, whereas reduced leaflet motion persisted or progressed in 20 (91%) of 22 patients who did not receive anticoagulation. These authors also found out that the mean aortic valve gradient at the time of the first CT scan was significantly higher in patients with reduced leaflet motion than in those without it. Patients with reduced leaflet motion were more likely to have aortic valve gradients < 20 mmHg than those with normal leaflet motion^[2]^. After detection of reduced leaflet motion, anticoagulation for three months was associated with a greater change in aortic valve mean gradients (decreased by 7.9 mmHg) than no anticoagulation.

Makkar et al.^[6]^ found out that reduced leaflet motion was detected among patients with multiple bioprosthesis types, including transcatheter aortic valve replacement (TAVR) and surgical bioprostheses. Therapeutic anticoagulation with warfarin, as compared with dual antiplatelet therapy, was associated with a decreased incidence of reduced leaflet motion^[6]^. In patients who were reevaluated with follow-up CT, restoration of leaflet motion was noted in all patients who were receiving anticoagulation.

Protocols for the diagnosis of subclinical BPVT have been published^[7]^. CT scan is by far the most accurate imaging tool for the diagnosis of subclinical thrombosis. Hypoattenuation associated with bioprosthetic leaflets, also described as hypoattenuated leaflet thickening, is the hallmark of subclinical leaflet thrombosis^[7,11]^. The hypoattenuating lesions involve the periphery and base of the leaflet and extend to varying degrees to the edges of the leaflet in the center of the bioprosthetic frame. Three-dimensional volume-rendered views may demonstrate abnormal leaflets visible as semilunar opacities in both systole and diastole^[11-15]^.

It has been established in recent studies that anticoagulation can reverse the hypoattenuation and restore normal leaflet motion with a significant impact on the mean aortic gradients measured by echocardiography^[2,6]^. Similar findings were observed on the follow-up of patients in the Portico trial^[2]^.

In an analysis of pooled data from the Portico IDE study, the RESOLVE and SAVORY registries, hypoattenuation and hypomotility were observed among various TAVR and surgical AVR devices in patients with aortic valve gradients within the normal range^[2]^. Therapeutic anticoagulation was associated with a reduced prevalence of hypoattenuation compared no therapy, suggesting a thrombotic mechanism. Moreover, leaflet mobility was fully restored after resolution of the phenomenon by warfarin, suggesting that reduced motion is a result rather than a cause of valve leaflet thrombosis.

The main finding of our study is that early anticoagulation after AVR is beneficial and associated with low risk of adverse events. We observed significantly lower mean and maximum transprosthetic gradients in patients who received at least three months of OA after AVR, both with warfarin and non-warfarin OA. Concomitantly, these patients also referred a significant improvement in NYHA class. The lower aortic valve gradient we found in patients receiving OA could probably be explained by the prevention of thrombus formation and, therefore, better leaflet mobility. Our results suggest that the benefit of OA in reducing mean gradient and improving NYHA class occurs mainly in patients with ≤ 21 mm bioprosthesis. A possible explanation for these findings stems from the fact that smaller bioprostheses are associated with more turbulence and, therefore, greater risk for valve thrombosis. Consequently, these patients could be candidates for OA. A randomized trial by our group is currently recruiting patients in order provide a definite answer.

### Limitations

This is a single-center study with a relatively small sample size and short-term follow-up. As a non-randomized study, selection bias is an inherent limitation of our results. Performance of CT scan would have contributed in the cause for the higher gradient in the non-OA group.

## CONCLUSION

We concluded that early anticoagulation in the postoperative period of AVR is associated with lower transprosthetic gradient and greater improvement in the NYHA functional class in the medium follow-up. This benefit may be restricted to patients with small bioprostheses.

**Table:** 

Authors' roles & responsibilities	
VD	Substantial contributions to the conception or design of the work; or the acquisition, analysis, or interpretation of data for the work; drafting the work or revising it critically for important intellectual content; final approval of the version to be published
PF	Substantial contributions to the conception or design of the work; or the acquisition, analysis, or interpretation of data for the work; drafting the work or revising it critically for important intellectual content; final approval of the version to be published
MJA	Substantial contributions to the conception or design of the work; or the acquisition, analysis, or interpretation of data for the work; drafting the work or revising it critically for important intellectual content; final approval of the version to be published
AF	Substantial contributions to the conception or design of the work; or the acquisition, analysis, or interpretation of data for the work; drafting the work or revising it critically for important intellectual content; final approval of the version to be published
DP	Substantial contributions to the conception or design of the work; or the acquisition, analysis, or interpretation of data for the work; drafting the work or revising it critically for important intellectual content; final approval of the version to be published
GS	Substantial contributions to the conception or design of the work; or the acquisition, analysis, or interpretation of data for the work; drafting the work or revising it critically for important intellectual content; final approval of the version to be published
